# Better Reporting of Scientific Studies: Why It Matters

**DOI:** 10.1371/journal.pmed.1001504

**Published:** 2013-08-27

**Authors:** The PLOS Medicine Editors

## Abstract

The *PLOS Medicine* Editors announce the launch of a Reporting Guidelines Collection to coincide with the Seventh International Congress on Peer Review and Biomedical Publication, to be held September 8–10, 2013, in Chicago.

*Please see later in the article for the Editors' Summary*

To coincide with the Seventh International Congress on Peer Review and Biomedical Publication to be held in Chicago from September 8 to 10, 2013 [1], *PLOS Medicine* is launching a new Reporting Guidelines Collection [2], an open access collection of reporting guidelines, commentary, and related research on guidelines from across *PLOS* journals. This collection is consistent with the goals of the Peer Review Congress: “to improve the quality and credibility of scientific peer review and publication and to help advance the efficiency, effectiveness, and equitability of the dissemination of biomedical information throughout the world” [2].

As early as 1990, Iain Chalmers, one of the founders of the Cochrane Collaboration, stated that, “Failure to publish an adequate account of a well-designed clinical trial is a form of scientific misconduct that can lead those caring for patients to make inappropriate treatment decisions.” [3]. Guidelines and checklists for reporting scientific studies are not just tick box exercises; rather, they help to improve the transparency and presentation of studies and, therefore, have the potential to improve the impact and implementation of scientific research.

*PLOS Medicine* has a strong history of promoting policies that aim to improve study design and transparency of reporting and publishing them in an open-access venue. We published our first reporting guideline – the STROBE (Strengthening the Reporting of Observational Studies in Epidemiology) Statement [4],[5] –more than 5 years ago. While the STROBE Statement was published concurrently with several other leading medical journals, critically, *PLOS Medicine* was the only open access journal to publish it at that time. For reporting guidelines to be useful, it is essential that they be widely disseminated, made freely available, and without restrictions on reuse. Since we published the STROBE Statement in 2007, there has been a shift toward making reporting guidelines more freely available; the EQUATOR (Enhancing the Quality and Transparency of Health Research; http://www.equator-network.org/) Network, launched in June 2008, provides freely accessible links to published guidelines.

To support *PLOS Medicine*'s aim of encouraging the highest possible standards in medical research and reporting, the journal launched “Guidelines and Guidance” in 2008, a new section within the Magazine that publishes reporting guidelines, research priorities, methodological issues, and other articles providing guidance on the conduct and reporting of research [6].

Reporting guidelines have evolved since the original CONSORT Statement was published in 1996 [7] as a minimum set of recommendations for reporting randomized controlled trials (RCT). The CONSORT Statement was updated in 2001 and 2010, and several extensions of the guidelines have been developed based on more specific study designs (e.g., CONSORT Statement for cluster-based RCTs [8]) or specific intervention types (e.g., acupuncture [9]). While RCTs provide the strongest evidence for clinical efficacy of interventions in a clinical setting and play a critical role in healthcare decision-making, they are not always feasible or ethical to conduct. Over time, reporting guidelines have been published for many other types of research that can also influence policy and practice, such as epidemiologic [4],[5], diagnostic [10], prognostic [11], and genetic risk prediction [12] studies. Similarly, extensions of the STROBE Statement have been developed as research fields emerge, such as for use by researchers conducting genetic association studies [13] or studies in molecular epidemiology [14].

An important development in evidence-based medicine has been the use of systematic reviews to synthesize the best quality research evidence relevant to a particular topic. One of the most frequently accessed and cited papers published in *PLOS Medicine* is the PRISMA Statement (Preferred Reporting Items for Systematic Reviews and Meta-Analyses) [15],[16], an evidence-based, minimum set of items for reporting of systematic reviews and meta-analyses. The PRISMA Statement has been endorsed by over 170 journals and includes a 27-item checklist and a four-phase flow diagram. On the *PLOS Medicine* website alone, it has over 100,000 views and has been cited 1,000 times [17].

Reporting guidelines have even been developed to improve abstract reporting for RCTs and systematic reviews, as extensions of CONSORT [18] and PRISMA [19], respectively. Abstracts are the first and often only part of an article that is read. Indeed, given that 50% of biomedical research is still behind a pay wall [20], the abstract is frequently the only part of the article that readers *can* access. Furthermore, about 40% of abstracts for RCTs have been shown to misrepresent or “spin” study findings [21], making it all the more critical that an abstract accurately represents the research findings.

While much of the focus of reporting guidelines has thus far been on health research, the animal research community is also developing reporting standards. The ARRIVE (Animal Research: Reporting *In Vivo* Experiments) guidelines were published in *PLOS Biology* in 2010 [22] and subsequently in 11 other journals. Recent efforts by the NC3Rs (National Centre for the Replacement, Refinement and Reduction of Animals in Research) have encouraged the adoption of the ARRIVE checklist. In an Editorial published in July 2013, *PLOS Medicine* announced a new requirement for the ARRIVE checklist for *in vivo* animal studies [23].

A growing body of evidence demonstrates improvements in the quality of reporting scientific studies associated with the publication of reporting guidelines; however, translation of the guidelines into practice remains a challenge. A systematic review, published by the Cochrane Group, observed that journal endorsement of the CONSORT Statement is associated with more complete reporting of trials in medical journals [24]. Other studies have reported improvements in the quality of reporting after publication of CONSORT guidelines for abstracts [25] and the PRISMA Statement [26]. In a randomized trial published in *BMJ*, conventional peer review plus review looking for missing items from reporting guidelines led to improvements in manuscript quality compared with conventional review [27]. However, studies also show that the quality of reporting overall remains suboptimal [24],[28], as not all journals endorse or enforce the use of reporting guidelines [29]–[31].

The EQUATOR Network website houses a comprehensive library of reporting guidelines for health research [32], of which our Collection is just a subset, as well as educational materials. The *PLOS Medicine* Editors strongly urge (and for specific articles types, require) authors, peer reviewers, and journal editors to use these freely available resources. Most reporting guidelines have checklists that can be submitted along with a manuscript to facilitate the peer review process by allowing editors and reviewers to quickly identify essential elements of how a study was conducted.

This new Reporting Guidelines Collection aims to highlight some of the many resources now available to facilitate the rigorous reporting of scientific studies, and to improve the presentation and evaluation of published studies. Transparency in research reporting should be integral to the dissemination of scientific research. The peer review process is a critical part of research and reporting guidelines provide a mechanism to help this process. While following reporting guidelines does not necessarily make the study better, this process does give readers the information to better judge the quality, and therefore the usefulness, of research. As online publication removes the space constraints of print, reporting should be complete and transparent, and reporting guidelines aid that process.

## Abbreviations

ARRIVE: Animal Research: Reporting *In Vivo* Experiments
CONSORT: Consolidated Standards of Reporting Trials
EQUATOR: Enhancing the Quality and Transparency of Health Research
PRISMA: Preferred Reporting Items for Systematic Reviews and Meta-Analyses
RCT: randomized controlled trial
STROBE: Strengthening the Reporting of Observational Studies in Epidemiology
